# P19 Antibiotic MOT: improving antimicrobial stewardship through nurse-led audit

**DOI:** 10.1093/jacamr/dlag102.025

**Published:** 2026-06-26

**Authors:** Luxy Baby, Nikala Astle-Wilson, Annie Joseph, Annette Clarkson, Sue Bowler

**Affiliations:** Nottingham University Hospitals NHS Trust, Nottingham, UK; Nottingham University Hospitals NHS Trust, Nottingham, UK; Nottingham University Hospitals NHS Trust, Nottingham, UK; Nottingham University Hospitals NHS Trust, Nottingham, UK; Nottingham University Hospitals NHS Trust, Nottingham, UK

## Abstract

**Objectives:**

To evaluate and enhance nurses’ and midwives’ roles in diagnostic and antimicrobial stewardship (AMS) by implementing a nurse-led audit and feedback programme that actively engages ward and senior nurses in AMS initiatives.

**Methods:**

A pilot audit was conducted using Tendable^®^. Question selection was informed by previous quality improvement work undertaken and were refined then validated by the AMS team following completion of the first four audits. The audit tool comprised 19 patient-level questions with 23 quality indicator sub-questions, alongside three open-ended questions. Wards were purposively selected to ensure broad representation of medical and surgical specialties. Data collection was undertaken by AMS nurses, with all responses anonymized. Results were summarized in a performance infographic and feedback to participating wards.

**Results:**

During the pilot phase, four inpatient wards across medical, surgical, and maternity areas were audited. Across these wards, 98% (*n*=44/45) of IV antibiotic doses were administered without omission, with a single appropriately documented missed IV dose on a surgical ward. Good practice in blood culture collection was observed in medical and surgical areas, including timely transport and loading of cultures on the incubator (100%, *n*=20/20) and blood cultures being taken prior to the first dose of IV antibiotics (95%, *n*=19/20). Medical areas also demonstrated good practice in obtaining two sets of blood cultures in 74% (*n*=14/19) of patients. Although no patients included in maternity areas met the criteria for blood culture collection at the time of audit, IV antibiotic prescribing was appropriate for 83% (*n*=5/6) of patients. However, several audit standards were not fully met. At the time of audit, only 67% (*n*=26/39) of patients in medical and surgical wards were appropriately receiving IV rather than oral antibiotics, and only 94% of required equipment was available in clinical areas to obtain and transport microbiology specimens. Documentation of IV-to-oral switch (IVOS) assessment was particularly poor, with only 3% (*n*=1/31) of nursing IVOS assessments recorded electronically and only one midwife documenting IVOS in maternity. In surgical areas, only 57% (*n*=4/7) of patients with an Early Warning Score (EWS) ≥5 had two sets of blood cultures taken, and in maternity areas, only 89% of required equipment was available.

**Conclusions:**

Examples of good AMS practices were observed across all areas; however, the audit also highlights several key areas requiring improvement, with different areas facing distinct challenges. This emphasizes the need for targeted ward-based interventions and ongoing support to embed AMS at ward-level. There was limited evidence of nursing staff prompting IVOS. As a result, the AMS team will focus on strengthening the role of the ward nurse in IVOS, while also considering other clinical roles that may be appropriate for initiating IVOS. Overall, the audit demonstrated the benefit of audit and feedback, to focus improvement resources supported by ward and senior nurse engagement.

**Next steps:**

Governance reporting of regular audit findings will be facilitated through dissemination of a performance infographic to senior nursing leads in the organization, via the Infection Prevention and Control Committee.

